# Hyperpolarized metabolic imaging of myocardial ischemia-reperfusion in a small-animal model at 9.4T

**DOI:** 10.1186/1532-429X-17-S1-O100

**Published:** 2015-02-03

**Authors:** Darach O h-Ici, Patrick Wespi, Julia Busch, Lukas Wissmann, Andreas Sigfridsson, Kilian Weiss, Daniel Messroghli, Sebastian Kozerke

**Affiliations:** 1 German Heart Institute, Berlin, Germany; 2 Biomedical Engineering, King's College London, London, UK; 3 Institute for Biomedical Engineering, ETH Zurich, Zurich, Switzerland

## Background

Dynamic hyperpolarization (DNP) of Carbon-13 (13C) allows in-vivo assessment of metabolic processes. The aims of the present study were to establish a living rat model of ischemia-reperfusion and to study the metabolic changes in the myocardium following short periods of coronary artery occlusion using intravenous injection of hyperpolarized 1-13C pyruvate.

## Methods

An inflatable balloon was secured around the left coronary artery of Sprague Dawley rats. 5-7 days after surgery rats were placed in a Bruker Biospec 9.4T small animal MR system. To enhance bicarbonate signal, animals received an iv glucose/potassium infusion. The tubing of the occluder was connected to extension tubing to allow occlusion while the animal remained in the bore of the MR system.

A custom-built multi-sample DNP polarizer (1) was used to polarize samples (25.4 μL [1-^13^C]-pyruvic acid and 13.5 mM trityl radical doped with 1.5 mM Dotarem). The rats were injected with 1.4 ml DNP solution at 4 time points: baseline, reperfusion directly after 15 minutes of coronary occlusion, after 30 minutes of reperfusion, after 60 minutes of reperfusion. A subgroup of rats underwent repeat imaging 1 week after ischemia. Metabolic data were acquired with a multiband pulse in combination with a multi-echo single-shot EPI readout (2). Hearts were removed and stained to delineate the area at risk (AAR), and for myocardial infarction.

## Results

**Figure 1:**
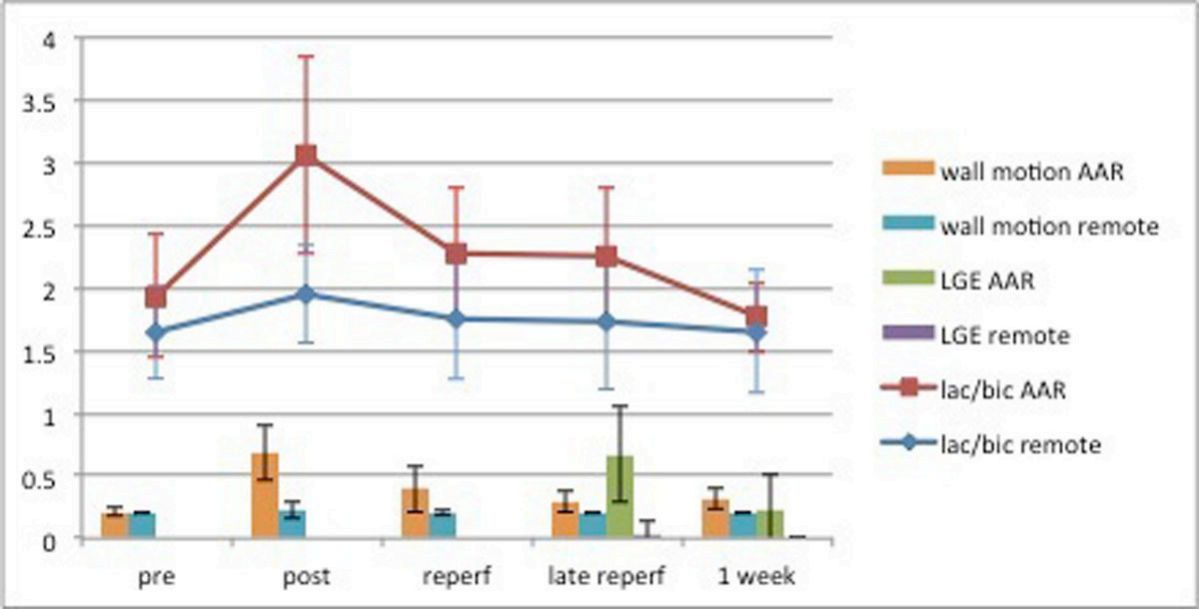
The lactate/bicarbonate ratio in the AAR was increased following 15 minutes of myocardial ischemia. It than decreased over the 60 minutes of measured reperfusion time. 1 week following ischemia this ratio was not significantly different from the remote area. The lower haft of the figure demonstrates that the AAR developed a localised hypokinesia following ischemia which recoverd over time, and that the AAR demonstrated acute injury as demonstrated by late gadolinium enhancement.

Data were successfully collected on 12 animals. By inflating and deflating the balloon, we could reversibly occlude/reopen the coronary artery. Occlusion led to hypokinesia of the anterior or anterior and lateral segments of the myocardium. The systolic function of the septum and inferior segments remained unchanged. Metabolic images of [1-^13^C]-pyruvate and its metabolites demonstrated that pyruvate was mainly localized in the heart chambers while [1-^13^C]-lactate and [1-^13^C]-bicarbonate were present in the myocardial wall. Immediately following reperfusion, the area-under-the-curve (AUC) of the lactate signal increased in the AAR, while the AUC of the bicarbonate signal decreased. This led to an increase in the lactate/bicarbonate ratio, which was unchanged in the remote area (Fig. 1). 7 animals were scanned at 1 week following ischemia-reperfusion. The lactate/bicarbonate ratio in the AAR was decreased compared to the 60-minute scan, and not significantly different from the remote area.

## Conclusions

Hyperpolarized [1-^13^C]-pyruvate identifies metabolic changes in the area-at-risk in a small animal model of ischemia-reperfusion. The myocardial metabolism in the AAR was found to be abnormal throughout the first 60 minutes following ischemia, but was seen to return to normal one week later.

## Funding

The work was supported by the Swiss National Science Foundation, grant #CR3213_132671/1. DOH is funded by a Sachmittelbeihilfe of the Deutsche Forschungsgemeinschaft (DFG).
